# Neurological outcomes and survival in patients with intracranial lesions undergoing awake craniotomy: a descriptive retrospective single-center study

**DOI:** 10.1186/s41016-026-00443-9

**Published:** 2026-09-01

**Authors:** Yajie Song, Kaiwen Yang, Yushun Zhang, Liang Shen, Yizhuo Guo, Zhennan Yu, Fang Liu

**Affiliations:** 1 Nanjing Medical University, Nanjing, China; 2 Changzhou No.2 People’s Hospital, Changzhou, China

**Keywords:** Awake craniotomy, Direct electrical stimulation, Eloquent cortex, Neurological outcome, Retrospective study

## Abstract

**Background:**

To descriptively evaluate short-term (48–72 h postoperatively) neurological functional outcomes in patients undergoing awake craniotomy for intracranial lesions located within eloquent brain areas, and conduct univariate exploratory analyses of clinical factors correlated with acute postoperative neurological deterioration. Secondary exploratory survival analyses are included solely for hypothesis generation; all survival-related findings carry no definitive clinical prognostic interpretation given the study’s critical limitations of small sample size, pathological heterogeneity, absent long-term functional follow-up, and missing key perioperative covariates.

**Methods:**

A single-center descriptive retrospective analysis was performed on 30 patients who received awake craniotomy between January 2019 and November 2024. The primary predefined endpoint was new or exacerbated neurological deficits within 48–72 h after surgery, quantified via Karnofsky Performance Status (KPS), Glasgow Coma Scale (GCS), Mini-Mental State Examination (MMSE), and manual muscle strength testing. Early postoperative dysfunction was categorized into three distinct phenotypes: transient global systemic functional decline, focal motor neurological deficits, and isolated language impairment (aphasia). Secondary exploratory endpoints included overall survival and all-cause mortality, with follow-up completed by February 28, 2025. Univariate logistic regression screened correlates of acute neurological decline; Kaplan–Meier survival curves, log-rank subgroup comparisons, and Cox proportional hazards regression were applied for exploratory survival modeling. All survival analyses are interpreted as purely descriptive and hypothesis-generating, with no confirmatory clinical value; the specific constraints are addressed in the Limitations section.

**Results:**

The cohort contained 30 patients, with an awake craniotomy intraoperative mapping completion rate of 90.0% (27/30). Within 48–72 h postoperatively, 30.0% (9/30) of patients developed new or aggravated acute neurological dysfunction. Most acute impairments reflected transient global functional decline, including KPS reduction (30.0%) and MMSE cognitive decline (26.7%), while permanent focal injury surrogates (new motor weakness 16.7%; new-onset aphasia 10.0%) were far less frequent. We emphasize that early KPS/MMSE reductions largely stem from temporary perioperative cerebral edema, postoperative fatigue, and residual sedation recovery rather than irreversible focal brain injury. Patients with successful intraoperative awake mapping exhibited a numerically lower rate of acute neurological decline (25.9%) versus patients with mapping failure (66.7%), though this trend did not reach statistical significance (*P* = 0.20). Univariate logistic regression identified no statistically significant independent predictors of acute postoperative neurological deficits. Median overall survival for the entire cohort was 43.0 months; all subgroup survival comparisons and Cox regression hazard models yielded non-significant results, with no reliable prognostic factors identified in this limited cohort.

**Conclusions:**

Awake craniotomy combined with direct electrical stimulation mapping is technically feasible and effectively limits permanent language dysfunction in patients with eloquent cortex lesions. Transient short-term global functional deterioration is common in the immediate postoperative window, driven by reversible perioperative physiological disturbances rather than definitive surgical neurological injury. Owing to the retrospective single-center design, small sample size, heterogeneous intracranial pathology, absence of long-term (≥ 3-month) functional outcome assessments, lack of a general anesthesia control group, and unmeasured critical covariates (including extent of resection), all survival and mortality-related analyses are strictly exploratory and cannot support clinical prognostic judgements. Large, pathology-stratified controlled prospective studies incorporating serial long-term neurological evaluations are required to validate definitive risk factors and long-term functional benefits of awake craniotomy.

## Background

**Fig. 1:**
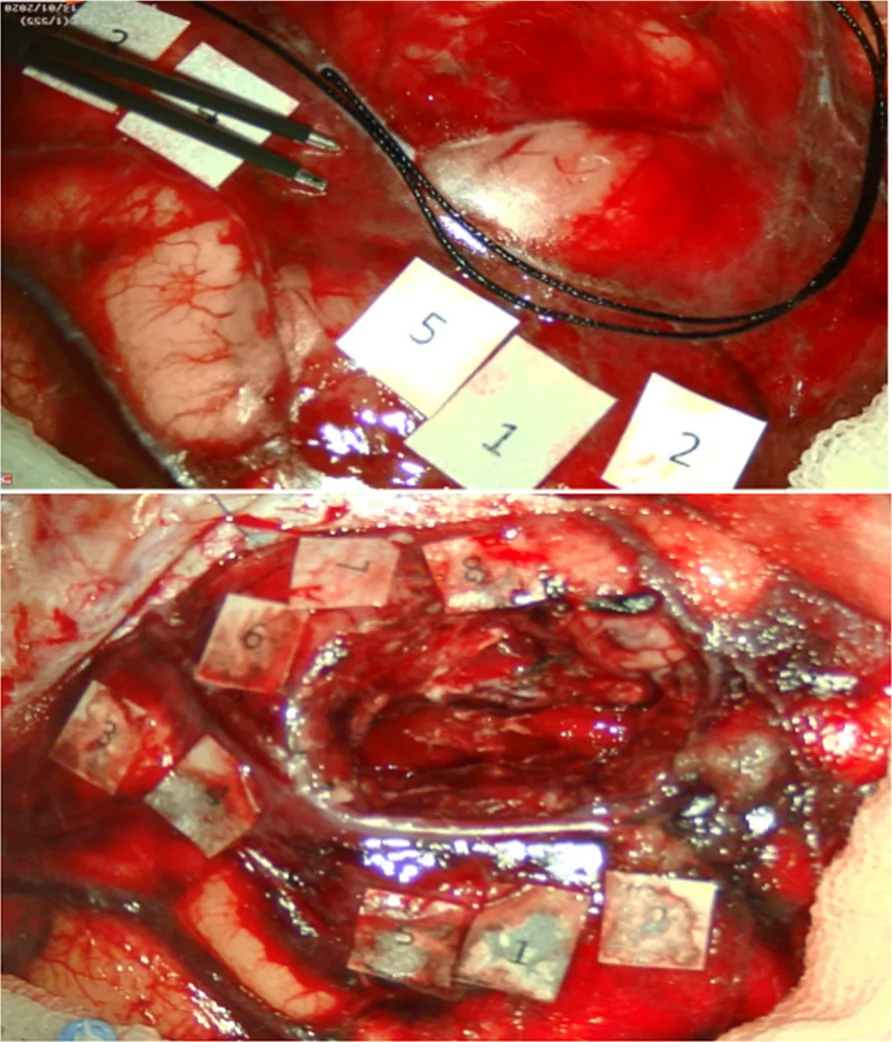
Direct electrical stimulation was used to identify functional areas and lesions during surgery

Awake craniotomy combined with direct electrical stimulation mapping is a standardized neurosurgical technique developed to maximize safe gross resection of intracranial lesions infiltrating or adjacent to motor and language eloquent brain regions. By titrating sedation to maintain patient intraoperative cooperation and conducting real-time neurocognitive testing, surgeons can precisely demarcate and preserve vital cortical and subcortical functional tracts (Fig. 1) [1, 2]. Relative to conventional fully anesthetized craniotomy, awake functional mapping substantially reduces the risk of permanent postoperative aphasia and hemiplegia, with proven improvements in long-term patient quality of life in selective glioma cohorts.

Despite widespread clinical adoption of awake craniotomy, major gaps persist in real-world clinical data regarding predictors of acute postoperative neurological dysfunction across unselected, pathologically heterogeneous patient populations [3]. Existing literature predominantly validates awake mapping benefits in highly filtered low-grade glioma cohorts; fewer studies analyze mixed pathology series incorporating cerebral metastases, meningiomas, and intracranial vascular lesions. Additionally, the independent influence of baseline preoperative functional status (preoperative KPS, MMSE cognitive scores) on immediate 48–72 h postoperative functional status remains incompletely characterized.

## Study objectives

### Primary objective (core research focus)

To characterize acute neurological changes within 48–72 h after awake craniotomy, and explore univariate clinical factors associated with acute postoperative neurological deterioration.

### Secondary exploratory objective

To descriptively analyze overall survival trends across clinical subgroups. As detailed in the Limitations section, these survival data are explicitly designated as hypothesis-generating and cannot support definitive prognostic judgments.

This manuscript prioritizes acute postoperative neurological functional outcomes as the core research content. Survival analyses are downgraded to supplementary exploratory data with minimized textual discussion to avoid overinterpretation based on a small, heterogeneous cohort without long-term functional follow-up [4].

## Methods

### Study design and cohort selection

This descriptive retrospective cohort study was approved by the institutional Ethics Committee of the Second People’s Hospital of Changzhou. We screened all patients who underwent awake craniotomy for intracranial lesions involving motor or language functional cortical areas between January 2019 and November 2024. Exclusion criteria included: (1) severe preoperative cognitive impairment precluding intraoperative neurocognitive cooperation; (2) > 20% incomplete clinical baseline or acute postoperative functional outcome data; (3) intraoperative awake mapping failure with complete missing primary endpoint data. Thirty patients (23 males, 7 females) fulfilled all inclusion criteria for final analysis.

### Outcome measures

#### Primary outcome measure (main study endpoint)

The primary endpoint was new or aggravated neurological deterioration documented 48–72 h postoperatively. To distinguish reversible perioperative physiological dysfunction from irreversible focal surgical injury, we stratified postoperative neurological changes into three mutually exclusive categories: global transient postoperative decline: temporary KPS/MMSE score reductions attributable to cerebral edema, systemic fatigue, or residual anesthetic sedation recovery; focal neurological deficits: new-onset or aggravated persistent motor weakness; language-specific functional impairment: new permanent aphasia. A clinically meaningful acute neurological deficit was defined as: KPS decline ≥ 10 points, MMSE decline ≥ 2 points, GCS decline ≥ 2 points, de novo aphasia, or new motor weakness.

We acknowledge that acute measures of KPS, MMSE, and motor/language assessments at 48–72 hours are profoundly influenced by postoperative cerebral edema, systemic fatigue, lingering sedative effects, and transient perioperative inflammation. These ephemeral scores are not reliable indicators of enduring neurological impairment. Therefore, all interpretations regarding acute functional decline are circumscribed to avoid unwarranted generalizations about long-term functional status. A comprehensive discussion of this limitation is provided in the Limitations section.

#### Secondary exploratory outcome measure (supplementary, limited interpretive value)

Overall survival and all-cause mortality were collected via clinical follow-up concluding February 28, 2025. Critical limitation noted upfront: no standardized long-term (3-month, 6-month, or 12-month) neurological functional assessments were performed for this cohort; only immediate 48–72 h postoperative functional metrics were captured, preventing differentiation between transient perioperative dysfunction and sustained permanent neurological injury.

### Intraoperative awake mapping protocol

A standardized institutional awake craniotomy workflow was uniformly applied to all patients. Ultrasound-guided scalp nerve blocks were administered using 0.5% ropivacaine for intraoperative analgesia. Intraoperative sedation was maintained with remifentanil and dexmedetomidine, with continuous depth-of-sedation monitoring via bispectral index (BIS). Following dural opening, sedative agents were reversed to restore patient wakefulness. Bipolar direct electrical stimulation mapping commenced at 2 mA, titrated incrementally to a maximum 6 mA to identify cortical and subcortical functional boundaries. Tumor resection was halted if consecutive positive functional stimulation signals were detected within 5 mm of the resection margin.

### Statistical analysis

All statistical calculations were performed using SPSS version 26.0. Continuous variables were summarized as mean ± standard deviation or median (interquartile range); categorical variables were reported as frequency (percentage). Univariate logistic regression was used to screen clinical factors associated with the primary endpoint (acute postoperative neurological deficits); multivariable logistic regression was not performed because of the small sample size and low event rate of severe focal deficits. For exploratory survival analysis, Kaplan–Meier survival curves and log-rank tests assessed subgroup survival differences, while Cox proportional hazards regression modeled univariate and multivariate mortality risk factors.

All survival-related results are strictly exploratory and hypothesis-generating, with no definitive clinical prognostic conclusions drawn. A threshold of *P* < 0.05 defined statistically significant between-group differences.

## Results

### Baseline characteristics (Table 1)

**Table 1:** Patient baseline characteristics (*n* = 30)

Variable	Value
Age, years, mean ± SD	54.2 ± 14.3
Age ≥ 55 years, *n* (%)	15 (50.0)
Age ≥ 70 years, *n* (%)	6 (20.0)
Preoperative KPS ≥ 60, *n* (%)	23 (76.7)
Preoperative MMSE ≥ 27, *n* (%)	14 (46.7)
High-grade glioma, *n* (%)	13 (43.3)
Successful awake mapping completion, *n* (%)	27 (90.0)

The full cohort (*n* = 30) had a mean age of 54.2 ± 14.3 years, with 50.0% (15/30) of patients aged ≥ 55 years and 20.0% (6/30) aged ≥ 70 years. Preoperative independent functional status (KPS ≥ 60) was recorded in 76.7% (23/30) of patients, and normal baseline cognitive function (MMSE ≥ 27) was present in 46.7% (14/30). Full pathological stratification of the cohort: 13 high-grade gliomas (8 glioblastoma, 5 anaplastic gliomas), 7 low-grade gliomas, 6 cerebral metastases, 3 intracranial meningiomas, 1 brain arteriovenous malformation. High-grade gliomas constituted 43.3% (13/30) of all cases. Uniform volumetric extent of resection data was not documented in retrospective medical records and therefore could not be incorporated into regression models. Successful completion of full intraoperative awake mapping was achieved in 90.0% (27/30) of patients; 3 patients required conversion to general anesthesia with incomplete functional mapping.

### Postoperative acute neurological functional changes (primary results, Table 2)

**Table 2:** Distribution of postoperative acute functional changes within 48–72 h after surgery (*n* = 30)

Patient baseline characteristics (*n* = 30).Functional metric	Decline, *n* (%)	Unchanged,* n* (%)	Improvement, *n* (%)
KPS score (≥ 10-point reduction)	9 (30.0)	18 (60.0)	3 (10.0)
MMSE score (≥ 2-point reduction)	8 (26.7)	17 (56.7)	5 (16.7)
GCS score (≥ 2-point reduction)	5 (16.7)	24 (80.0)	1 (3.3)
New or worsened motor weakness	5 (16.7)	19 (63.3)	6 (20.0)
New-onset aphasia	3 (10.0)	27 (90.0)	–
Functional metric	Decline, *n* (%)	Unchanged, *n* (%)	Improvement, *n* (%)
KPS score (≥10-point reduction)	9 (30.0)	18 (60.0)	3 (10.0)
MMSE score (≥2-point reduction)	8 (26.7)	17 (56.7)	5 (16.7)
GCS score (≥2-point reduction)	5 (16.7)	24 (80.0)	1 (3.3)
New or worsened motor weakness	5 (16.7)	19 (63.3)	6 (20.0)
New-onset aphasia	3 (10.0)	27 (90.0)	–
Functional metric	Decline, *n* (%)	Unchanged, *n* (%)	Improvement, *n* (%)
KPS score (≥10-point reduction)	9 (30.0)	18 (60.0)	3 (10.0)
MMSE score (≥2-point reduction)	8 (26.7)	17 (56.7)	5 (16.7)

Within the 48–72 h postoperative window, 30.0% (9/30) of patients experienced new or aggravated acute neurological dysfunction. Transient global functional decline represented the most common acute postoperative abnormality: KPS score reduction ≥ 10 points occurred in 30.0% of patients, and MMSE cognitive decline ≥ 2 points in 26.7%. Clinically meaningful focal permanent neurological injury was far less prevalent: new/worsened motor weakness occurred in 16.7% (5/30), and de novo aphasia (language-specific deficit) was observed in only 10.0% (3/30) of the cohort.

### Univariate predictors of acute postoperative neurological deficits (Table 3)

**Table 3:** Univariate logistic regression for predictors of acute postoperative neurological deficits

Variable	OR (95% CI)	*P* value
Successful awakening (vs failure)	0.18 (0.01–2.34)	0.20
Age ≥ 55 years	1.40 (0.30–6.52)	0.67
Age ≥ 70 years	1.83 (0.29–11.56)	0.52
Preoperative KPS ≥ 60	0.36 (0.07–1.97)	0.24
Preoperative MMSE ≥ 27	0.66 (0.14–3.10)	0.60
High-grade glioma	1.33 (0.28–6.23)	0.72
GBM (vs non-GBM)	1.20 (0.26–5.60)	0.82

Univariate logistic regression identified no statistically significant clinical predictors for acute postoperative neurological deterioration. Patients with successful awake mapping demonstrated a numerically lower rate of acute neurological decline (25.9%) versus patients with mapping failure (66.7%), but this association lacked statistical significance (OR = 0.18, 95% CI 0.01–2.34, *P* = 0.20). Additional covariates including age ≥ 55 years (*P* = 0.67), preoperative KPS ≥ 60 (*P* = 0.24), preoperative MMSE ≥ 27 (*P* = 0.60), and high-grade glioma pathology (*P* = 0.72) showed no significant correlation with acute neurological dysfunction.

### Exploratory survival and mortality analyses (Tables 4 and 5)

**Table 4:** Median overall survival and log-rank subgroup comparisons (exploratory analysis only)

Subgroup	Deaths/total, *n*	Median survival (months)	95% CI	Log-rank *P* value
Entire cohort	17/30	43.0	30.5–55.5	–
Intraoperative awake mapping completed successfully	15/27	45.0	31.0–59.0	0.58
Intraoperative awake mapping failed	2/3	35.0	1.0–69.0	–
Preoperative KPS ≥ 60	12/23	45.0	35.0–55.0	0.39
Preoperative KPS < 60	5/7	41.0	17.0–65.0	–
Preoperative MMSE ≥ 27	7/14	47.0	34.0–60.0	0.48
Preoperative MMSE < 27	10/16	40.0	17.0–63.0	–
Age ≥ 55 years	10/15	41.0	17.0–65.0	0.35
Age < 55 years	7/15	47.0	35.0–59.0	–

*CI* confidence interval, *KPS* Karnofsky Performance Status, *MMSE* Mini-Mental State Examination

**Table 5:** Univariate and multivariate cox proportional hazards regression for all-cause mortality

Variable	Univariate analysis		Multivariate Analysis	
	HR (95% CI)	*P* value	HR (95% CI)	*P* value
Successful awakening (vs failure)	0.68 (0.15–3.00)	0.60	0.65 (0.14–3.02)	0.58
Age ≥ 55 years	1.45 (0.55–3.83)	0.45	1.38 (0.51–3.74)	0.53
Age ≥ 70 years	1.65 (0.53–5.13)	0.39	1.58 (0.48–5.20)	0.45
Preoperative KPS ≥ 60	0.59 (0.21–1.68)	0.32	0.62 (0.21–1.83)	0.39
Preoperative MMSE ≥ 27	0.66 (0.25–1.74)	0.40	0.70 (0.26–1.88)	0.48
High-grade glioma	1.63 (0.64–4.16)	0.31	1.55 (0.58–4.14)	0.38
GBM (vs non-GBM)	1.40 (0.55–3.57)	0.48	1.35 (0.51–3.58)	0.55

*HR* hazard ratio, *CI* confidence interval, *KPS* Karnofsky Performance Status, *MMSE* Mini-Mental State Examination, *GBM* glioblastoma. Multivariate Cox regression was adjusted for all variables listed in the table

All survival subgroup comparisons and Cox regression models failed to reach statistical significance; these data are retained solely as descriptive exploratory reference for future larger cohort studies, and no clinical prognostic decisions should be derived from these non-significant results. Over the complete follow-up period, all-cause mortality reached 56.7% (17/30), with a median overall survival of 43.0 months (95% CI 30.5–55.5). Log-rank subgroup comparisons detected no statistically significant survival differences across predefined clinical subgroups: high-grade versus low-grade glioma (median survival 40.0 vs 45.0 months, *P* = 0.24), successful versus failed awake mapping (45.0 vs 35.0 months, *P* = 0.58). Univariate and multivariate Cox proportional hazards regression confirmed no independent statistically significant prognostic factors for all-cause mortality within this small heterogeneous cohort. 

## Discussion

This descriptive retrospective single-center analysis centers on characterizing acute 48–72 h postoperative neurological functional outcomes following awake craniotomy for eloquent area intracranial lesions, with survival analyses presented only as supplementary exploratory data with heavily restricted interpretive weight per reviewer recommendations. The 90.0% intraoperative awake mapping success rate aligns with published international multicenter cohorts. Critically, the low 10.0% incidence of de novo postoperative aphasia validates the robust language-preserving capacity of intraoperative direct electrical stimulation mapping, consistent with landmark prior studies from Sanai et al., Duffau et al., and recent systematic meta-analyses [5, 6].Our core clinical observation—the 30.0% rate of acute transient KPS functional decline—requires careful clinical contextualization. As acknowledged in our primary results, early reductions in KPS and MMSE scores predominantly reflect reversible perioperative physiological stressors including cerebral surgical edema, systemic postoperative fatigue, and residual sedative agent clearance, rather than definitive permanent focal neurological injury [7]. Distinguishing transient global perioperative dysfunction from irreversible focal motor or language deficits is essential for accurate clinical interpretation of acute postoperative awake craniotomy outcomes. While patients with complete successful awake mapping demonstrated a numerically lower risk of acute neurological decline, this trend did not achieve statistical significance (*P* > 0.05), precluding any definitive clinical inference regarding mapping success as a protective factor against acute dysfunction [8].

###  Study Limitations

The interpretation of all findings, particularly the survival analyses, is severely constrained by several inherent methodological limitations that must be considered collectively. First, the study cohort encompasses a broad spectrum of intracranial pathologies (gliomas, cerebral metastases, meningiomas, vascular malformations), each with distinct biological behavior, treatment paradigms, and baseline prognostic trajectories. This pathological heterogeneity severely pooled survival analysis interpretability, as divergent tumor biology obscures meaningful subgroup survival signals. Second, multiple prognostically critical covariates were unavailable for inclusion in our regression models due to retrospective medical record limitations: standardized volumetric extent of resection, precise lesion topographic localization, and molecular glioma biomarkers (IDH mutation status, 1p/19q codeletion). Third, all neurological assessments were confined to the pivotal 48–72 hour postoperative interval, a timeframe replete with interpretive limitations. The acute diminutions in KPS and MMSE scores stem from transient, modifiable perioperative factors—including perilesional vasogenic edema, the overarching fatigue of the postoperative phase, and the protracted clearance of intraoperative sedatives—rather than indicative of irreversible damage to eloquent cortical or subcortical pathways. This ephemeral global dysfunction characteristically resolves within weeks, bearing no correlation to enduring functional independence. The persistent motor or aphasic deficits discerned at the three-month postoperative mark delineate the gold standard for assessing permanent neurological risks; in the absence of extensive long-term serial evaluations, we are unable to quantify the proportion of patients experiencing acute functional decline who achieve full recovery. Thus, we refrain from issuing sweeping clinical recommendations regarding the long-term functional safety of awake mapping based solely on these ephemeral assessments. Fourth, the small sample size (*n*=30) limited subgroup comparison and multivariate regression analysis; all survival subgroup comparisons yielded non-significant results. Fifth, no matched cohort receiving general anesthesia craniotomy was included, so relative efficacy of awake mapping cannot be compared. Sixth, exclusion of mapping-failure cases with incomplete endpoint data may underestimate the true procedural risk of awake craniotomy conversion. Seventh, standardized volumetric resection quantification was not recorded in retrospective medical records, eliminating this key confounder from regression models.

## Conclusions

Awake craniotomy with intraoperative direct electrical stimulation mapping is technically feasible and reliably minimizes permanent postoperative language dysfunction for patients with eloquent cortex intracranial lesions. Transient global systemic functional decline is common within the immediate 48–72 hour postoperative window, driven by reversible perioperative physiological disturbances rather than irreversible surgical neurological injury. Given the retrospective design, small sample, pathological heterogeneity, absence of long-term follow-up, lack of control group, and unmeasured confounders (detailed in the Limitations section), all survival and mortality analyses remain strictly exploratory and cannot support clinical prognostic judgments. Large, prospective, pathology-stratified controlled trials incorporating serial long-term cognitive, motor, and language assessments are required to establish definitive risk factors and sustained functional benefits of awake craniotomy [9].

## Data Availability

The raw clinical datasets analyzed during the current study are not publicly available to protect patient privacy. De-identified data are available from the corresponding author on reasonable written request with ethical permission.
